# Intestinal permeability in patients with IgA nephropathy and other glomerular diseases: an observational study

**DOI:** 10.1007/s40620-022-01454-2

**Published:** 2022-09-15

**Authors:** Claudia Seikrit, Judith I. Schimpf, Stephanie Wied, Eleni Stamellou, Ana Izcue, Oliver Pabst, Thomas Rauen, Kaatje Lenaerts, Jürgen Floege

**Affiliations:** 1 Division of Nephrology and Clinical Immunology, RWTH Aachen University, Pauwelsstr. 30, 52057 Aachen, Germany; 2 Department of Internal Medicine III, Nephrology and Dialysis, Feldkirch Academic Teaching Hospital, Feldkirch, Austria; 3 Department of Medical Statistics, RWTH Aachen University, Aachen, Germany; 4 Department of Molecular Medicine, RWTH Aachen University, Aachen, Germany; 5 Department of Surgery, NUTRIM School for Nutrition and Translational Research in Metabolism, Maastricht University, Maastricht, The Netherlands

**Keywords:** IgA nephropathy, Gut-kidney axis, Intestinal permeability, Multi sugar test

## Abstract

**Background:**

A dysregulated ‘gut-kidney axis’ may contribute to immunoglobulin A nephropathy (IgAN). We studied whether IgAN patients have disturbed intestinal permeability.

**Methods:**

In a prospective, cross sectional, pilot study we assessed intestinal permeability in 35 IgAN patients, 18 patients with non-IgAN glomerulonephritides (GNs) and 19 healthy controls. After an overnight fast, trial participants ingested a multi-sugar solution and samples were obtained from 0 to 2, 2 to 5- and 5 to 24-h urine portions. Urinary sugar concentrations were quantified using isocratic ion-exchange high performance liquid chromatography. Indices of small intestinal permeability (0–2-h lactulose/L-rhamnose (L/R) ratio), distal small intestinal and proximal colonic permeability (2–5-h sucralose/erythritol (S/E) ratio) and colonic permeability (5–24-h sucralose/erythritol (S/E) ratio) were evaluated. Associations between groups and indices of intestinal permeability were investigated by a linear mixed model.

**Results:**

Small intestinal permeability (0–2 h L/R-ratio) was significantly increased in patients with glomerular diseases versus healthy controls. More precisely, increased small intestinal permeability was exclusively noted in non-IgAN GN patients, whereas IgAN patients exhibited a trend towards elevated small intestinal permeability. In total, 54% of patients with IgAN and 67% of non-IgAN GN patients had increased small intestinal permeability. Neither distal small intestinal and proximal colonic permeability nor colonic gut permeability indices (i.e., 2–5 h and 5–24 h S/E ratios) were significantly different between controls and any of the GN patient groups.

**Conclusion:**

The present single center pilot study suggests that disturbed intestinal permeability is common in patients with glomerular diseases and is not specific for IgAN.

**Trial registration number:**

German Clinical Trials Register DRKS00021533, Date: 24.04.2020.

**Graphical abstract:**

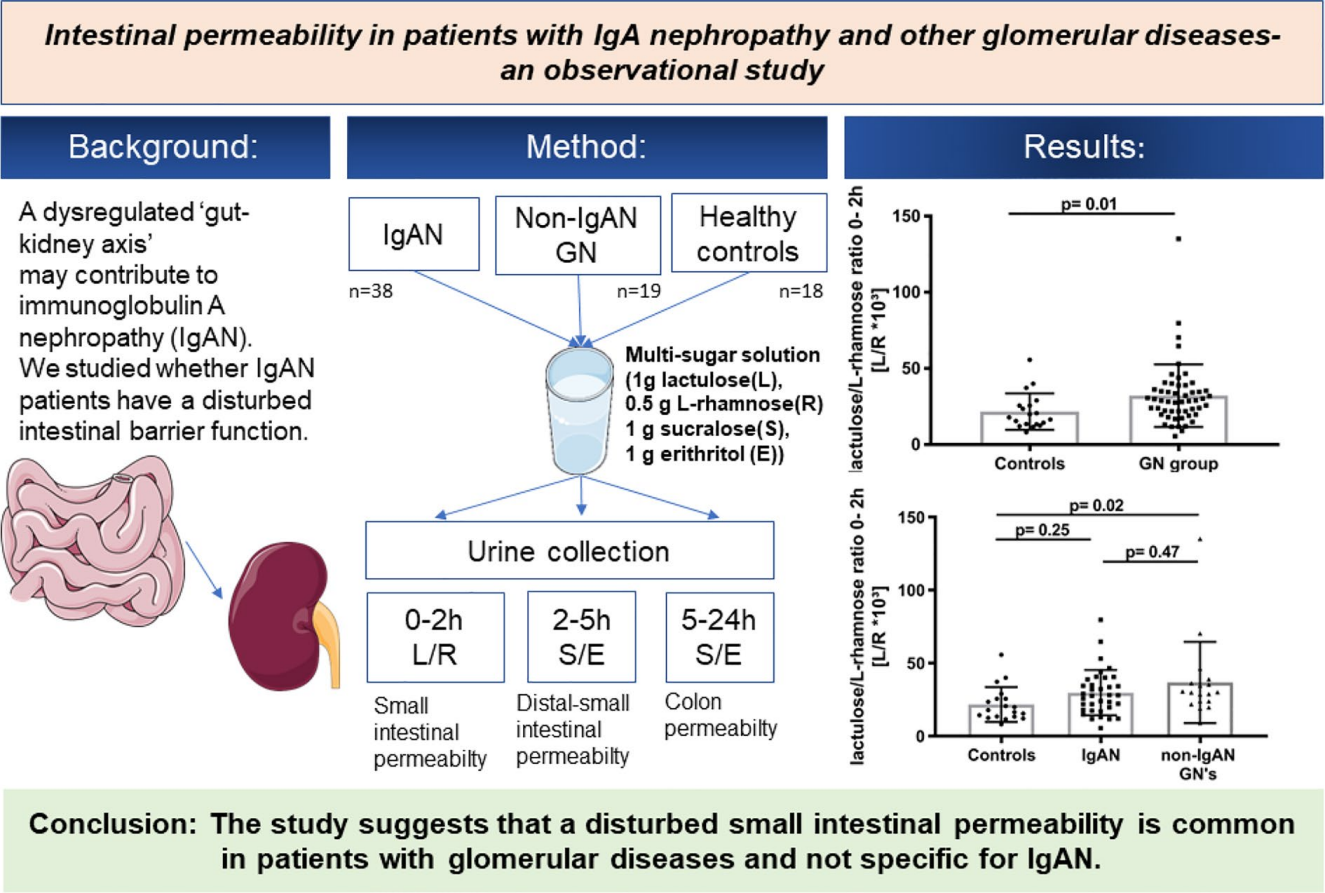

## Introduction

IgA nephropathy (IgAN) is the most common primary glomerular disease worldwide [1]. Since its first description in 1968 by Jean Berger and Nicole Hinglais it is clear that clinical presentation of IgAN is highly variable, ranging from mild forms with only minor urinary findings and preserved renal function to cases with rapid progression to end-stage renal disease (ESRD) [2]. Research into the pathophysiology of IgAN has been hampered by the lack of suitable animal models since the human IgA system is relatively unique. However, extensive research in patients has identified numerous central pathogenic contributors including genetic, immunological and environmental factors [1, 3, 4].

The role of a disturbed intestinal barrier function in the pathophysiology of IgAN has been intensively discussed. In 2014, a genome wide association study (GWAS) in more than 7500 European and East Asian IgAN patients identified several susceptibility genes that are implicated in intestinal immunity, pointing out the possible implication of the mucosal immune system in the pathophysiology of IgAN [5]. An increase in intestinal permeability is one of the indicators of disturbed mucosal barrier function. A damaged intestinal barrier in patients with IgAN could potentially give systemic access to multiple environmental and microbial antigens that are presented to the mucosal immune system. Immune processes activated by these antigens might in turn lead to enhanced production of undergalactosylated IgA (Gd-IgA1) provoking disease activation or flares. Several studies have investigated intestinal permeability in IgAN patients in the past [6–10], but due to different experimental approaches and missing disease controls in some of the studies these findings were not conclusive. Nevertheless, recently a therapeutic approach targeting the ‘gut-kidney axis’ was pursued in the *Nefigan* trial [11]. A targeted-release formulation of budesonide, a drug designed to be largely effective in the distal ileum, led to a dose-dependent and sustained reduction of proteinuria in IgAN patients and prevented estimated glomerular filtration rate (eGFR) loss over one year. This strongly indicates that local regulation of the mucosal immune system is important in IgAN [12].

In the present study, we aim to further characterize intestinal permeability in patients with IgAN and to compare it to patients with non-IgAN glomerulopathies. Unlike former studies, where either the radioactive chromium-51 labeled ethylenediamine tetraacetic acid (^51^Cr-EDTA) assay or two-sugar assays (i.e. cellulose/mannitol) were employed, we used a novel multi-sugar assay allowing to additionally distinguish between small intestinal and whole gut permeability [13, 14] and to investigate whether impaired intestinal permeability is specific for IgAN and whether it associates with progression markers such as proteinuria and renal function.

## Methods

### Study population

Between June 2017 and May 2019 patients with IgAN, patients with other glomerulonephritides (non-IgAN GNs) and healthy individuals were recruited at the Division of Nephrology of the RWTH Aachen University Hospital. Every patient who presented at our center during the above-mentioned period and met the inclusion and exclusion criteria was invited to participate in the study. All study participants were at least 18 years of age. Renal diagnoses had been confirmed by prior kidney biopsy that was not specifically performed for the purpose of enrollment into the present study. Patients with a history of chronic gastrointestinal diseases, signs of acute infection, diabetes mellitus, end-stage renal disease, or intake of non-steroidal anti-inflammatory drugs (NSAIDs) during the last 48 h prior to the study, as well as pregnant and breast-feeding patients were excluded. Written, informed consent was obtained from all study participants prior to the study. The study protocol was approved by the local ethics committee (EK 238/16), and was registered in the German Clinical Trials Register (DRKS00021533).

### Study design

After an overnight fast, baseline blood and urine samples were taken and serum creatinine, C-reactive protein levels, a red blood cell count and baseline proteinuria were determined. Additionally, pregnancy was excluded in females by measuring human choriongonadotropin (ß-HCG) levels. Subsequently, trial participants ingested a four-sugar-mixture solubilized in 200 ml of water. The mixture contained 1 g of lactulose (Centrafarm BV, Etten-Leur, The Netherlands), 0.5 g of l-rhamnose (Danisco A/S, Copenhagen, Denmark), 1 g of sucralose (Brenntag AG, Mülheim, Germany) and 1 g of erythritol (Cargill Europe, Mechelen, Belgium). After ingestion, all participants collected urine for 24 h in three separate fractions: 0–2 h, 2–5 h and 5–24 h. During the first 5 h of urine collection, participants were asked to refrain from any food or drinks, except for water (max. 500 ml water per hour). No additional blood samples were taken. After 5 h the participants left the study center and were allowed to consume food but were asked to refrain from ingestion of foods containing sweeteners during the collection period. Twenty-four hours after sugar ingestion, patients returned to our center to provide the 5–24 h urine fraction. For sugar analyses all urine samples were centrifuged at 4 °C and 3,500 rpm for 10 min prior to freezing at − 80 °C for storage.

### Sugar measurement

Sugar concentrations were determined by isocratic ion-exchange High Performance Liquid Chromatography with mass spectrometry as described by van Wijck et al. [13, 14]. The ratio between lactulose and l-rhamnose (0–2 h L/R) in the urine fraction collected within the first 2 h after oral intake was considered to reflect small intestinal permeability. Bacteria which are more abundantly present in the distal gastrointestinal (GI) tract are able to metabolize sugars as lactulose and rhamnose [15], whereas sucralose (S) and erythritol (E) remain unaffected. Hence, the S/E ratio measured in the 2–5-h urine collection reflects the permeability of the distal small intestine and proximal colon (2–5 h S/E). Colon permeability is reflected by S/E ratio in the 5–24-h urine collections (5–24 h S/E).

### Statistical analyses

Baseline characteristics are presented as medians and interquartile range or percentages. Ratios for small intestinal permeability, distal small intestinal and proximal colonic permeability and colonic permeability were defined as outcome parameters. Proteinuria, eGFR, urinary volume, age and gender were presumed as potential confounders/effect modifiers. The three study groups were defined as predictors. Each of the three ratios for the individual intestinal compartments between the three study groups (i.e. IgAN patients, patients with non-IgAN GNs and healthy controls) were analyzed using a linear mixed model (PROC Mixed in SAS) adjusting for proteinuria, eGFR, urinary volume, age and gender. The difference between the healthy controls and the pooled group of all GNs (i.e. IgAN and non-IgAN GNs together, referred to as the GN group) was calculated by linear contrasts. To improve the model fit, each ratio was logarithmically transformed after visual examination of the residual plots. The relationship between each of the three permeability ratios and either eGFR or proteinuria was investigated by Spearman correlations. Differences between the frequencies of patients with 0–2 h L/R values over the 95% confidence interval of healthy controls were evaluated using contingency tables and Fisher’s exact test. *P*-values < 0.05 were considered statistically significant. Statistical analyses were performed using SAS (version 9.4, SAS Institute Inc, Cary, NC, USA) and figures were created with Graph Pad Prism (version 7).

## Results

### Baseline demographics

A total of 35 IgAN patients, 18 non-IgAN GN patients and 19 healthy individuals were included in the trial. Within the IgAN group, 19 patients had a proteinuria of less than 0.5 g/day, eleven patients were between 0.5 and 1 g/day, and five patients were over 1 g/day. Some of the IgA patients already had significantly impaired renal function independent from the degree of proteinuria. Two IgAN patients received low-dose corticosteroids.

The study group of non-IgAN GNs comprised six patients with minimal change disease, three patients with focal-segmental glomerulosclerosis, three patients with membranous GN, four patients with lupus nephritis, two patients with idiopathic membranoproliferative GN and one patient with granulomatosis with polyangiitis. Fifteen of these patients were on immunosuppression at the time of study inclusion. In detail, five patients were being treated with corticosteroid monotherapy, six patients with a combination of corticosteroids and calcineurin inhibitors (CNIs), two patients with corticosteroids and mycophenolate acids, and two patients with corticosteroids, CNIs and mycophenolic acids. Three patients did not receive immunosuppressive therapy.

Patients with IgAN were older than the other study participants and there were more males in the IgAN group as compared to the two other groups (Table 1). Patients with IgAN exhibited a lower average eGFR level as compared to patients with non-IgAN GNs. These differences were blunted when IgAN and non-IgAN GN patients were grouped according to their chronic kidney disease (CKD) stage, i.e., CKD 1–2 versus CKD 3–4 (Table 2).

**Table 1:** Baseline demographics of the study cohort

	Healthy controls (*n* = 19)	IgAN (*n* = 35)	Non-IgAN GNs (*n* = 18)
Age (yrs)	37 (28–48)	51 (29–61)	42 (30–47)
Males (%)	52.63	88.57	44.44
Blood pressure (mmHg)			
Systolic	122 (110–130)	122 (113–132)	117 (111–129)
Diastolic	77 (70–80)	75 (70–83)	76 (71–80)
BMI (kg/m^2^)	21.94 (20.83–26.22)	26.67 (24.31–30.86)	27.15 (24.34–28.74)
Current smoker (%)	21.05	17.14	33.33
Immunsuppression (*n*/total)	0/0	2/35	15/18
eGFR (ml/min/1.73 m^2^)	100.70 (87.90–114.2)	54.50 (36.40–82.60)	86.45 (51.80–101.10)
Serum creatinine (mg/dl)	0.86 (0.78–0.98)	1.50 (1.09–1.97)	0.96 (0.73–1.62)
Proteinuria (g/d)	0.10 (0.09–0.13)	0.46 (0.18–0.86)	0.19 (0.11–0.55)

Values are presented as medians and Interquartile Range (IQR) or percentages

*BMI* body mass index, *eGFR* estimated glomerular filtration rate

**Table 2:** Baseline demographics of the study cohort grouped by CKD stage

	CKD stage 1–2 (*n* = 29)		CKD stage 3–4 (*n* = 24)	
	IgAN (*n* = 16)	Non-IgAN GNs (*n* = 13)	IgAN (*n* = 19)	Non-IgAN GNs (*n* = 5)
Age (yrs)	37.50 (25.0–54.5)	40 (30.0–47.0)	57.00 (46.0–64.0)	44.00 (34.0–45.0)
Males (%)	87.50	38.46	89.47	60.00
Blood pressure (mmHg)				
Systolic	115 (109–128)	115 (111–121)	127 (117–139)	126 (114–143)
Diastolic	72 (70–76)	75 (74–77)	82 (71–86)	78 (71–97)
BMI (kg/m^2^)	26.21 (24.23–30.22)	27.55 (24.34–28.74)	27.71 (24.98–30.93)	26.88 (25.99–27.42)
Current smoker (%)	6.25	38.46	26.32	20.00
Immunosuppression (*n*/total)	1/16	10/13	1/19	5/5
eGFR (ml/min/1.73 m^2^)	83.80 (70.05–96.55)	97.70 (84.6–105.1)	36.50 (22.50–49.40)	45.40 (36.10–50.90)
Creatinine (mg/dl)	1.09 (0.99–1.16)	0.92 (0.72–0.98)	1.93 (1.64–2.99)	1.73 (1.70–1.96)
Proteinuria (g/d)	0.38 (0.15–0.79)	0.19 (0.14–0.55)	0.47 (0.19–0.95)	0.18 (0.10–0.31)

Values are presented as medians and Interquartile Range (IQR) or percentages. CKD, chronic kidney disease

*BMI* body mass index, *eGFR* estimated glomerular filtration rate

### Intestinal permeability indices

**Fig. 1:**
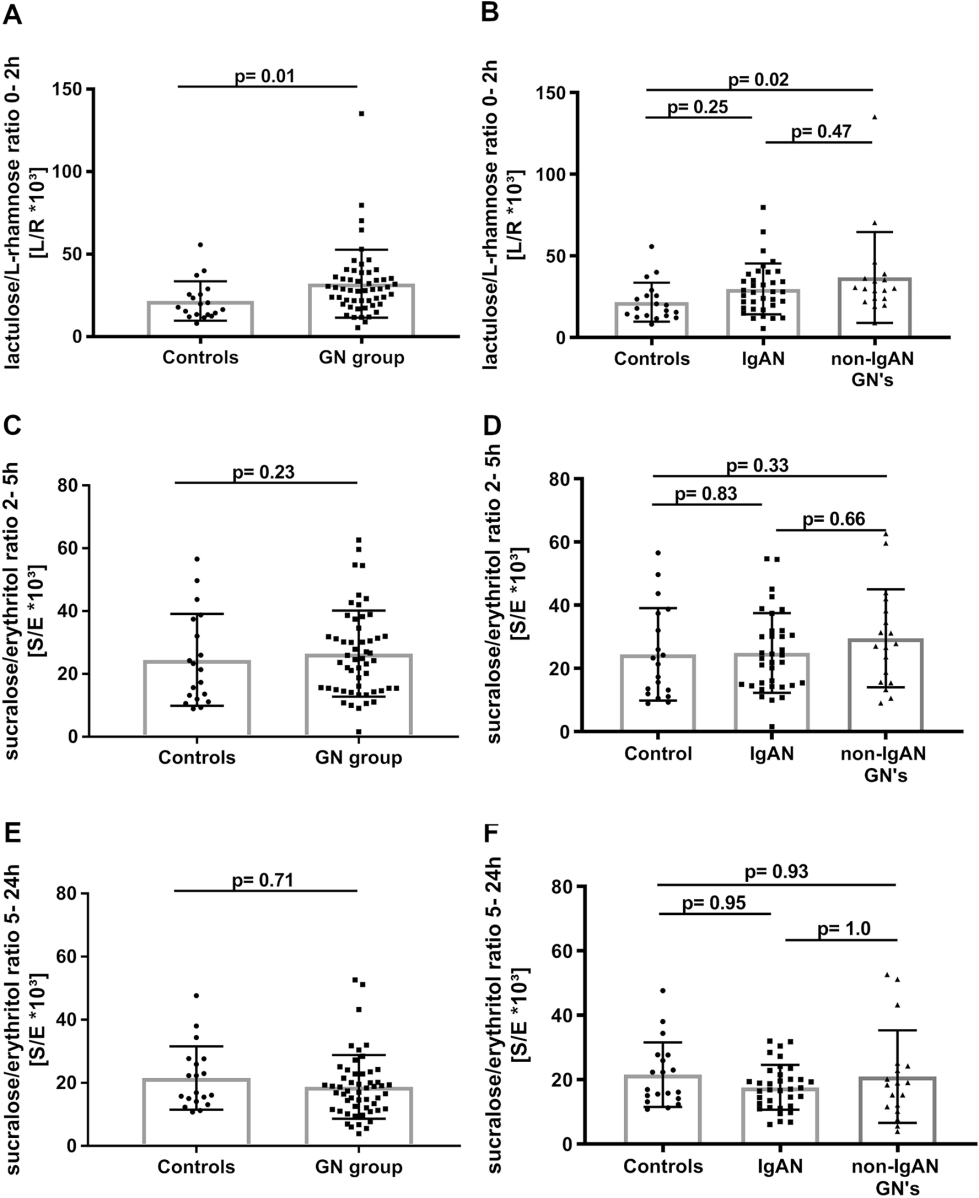
Gut permeability indices of the three study groups. **A** The 0–2 h-L/R ratio was significantly elevated in patients with glomerular diseases (i.e., GN group: Pool of IgAN and non-IgAN GN data) compared to healthy controls (adjusted for age, gender, eGFR and proteinuria).** B** Patients with non-IgAN GNs exhibited a higher 0–2 h-L/R ratio, pointing to significantly increased small intestinal permeability compared to healthy controls (multivariable analysis). Patients with IgAN had no significant increase in the 0–2 h-L/R ratio versus healthy controls. **C** The 2–5 h-S/E ratio did not significantly change between the pooled GN-group and healthy controls. **D** No significant changes of the 2–5 h-S/E ratio could be seen between healthy controls, IgAN and non- IgAN GN patients.** E** There was no significant difference of the 5–24 h-S/E ratio between the patients with glomerular diseases compared to healthy controls.** F** The 5–24 h-S/E ratio was not significantly changed between the three groups. 0–2 h-L/R ratio, 0–2-h lactulose/L-rhamnose ratio; 2–5 h-S/E ratio, 2–5-h sucralose/erythritol (2–5 h-S/E) ratio; 5–24 h-S/E ratio, 5–24-h sucralose/erythritol ratio

Small intestinal permeability (as assessed by the 0–2 h L/R ratio) was significantly increased in patients with glomerular diseases (GN group), i.e. the pooled group of patients with IgAN and non-IgAN GNs, as compared to healthy individuals (*p* = 0.01; Fig. 1A). Within the GN group, patients with non-IgAN GNs exhibited significantly higher small intestinal permeability as compared to controls (*p* = 0.02; Fig. 1B), whereas small intestinal permeability was not significantly different between IgAN patients and both control groups (*p* = 0.47 and *p* = 0.25, respectively). Neither the distal small intestinal and proximal colonic permeability nor the colonic permeability ratios (i.e. 2–5 h and 5–24 h S/E ratios) were different between healthy controls and any of the patient groups (Fig. 1C–F).

**Fig. 2:**
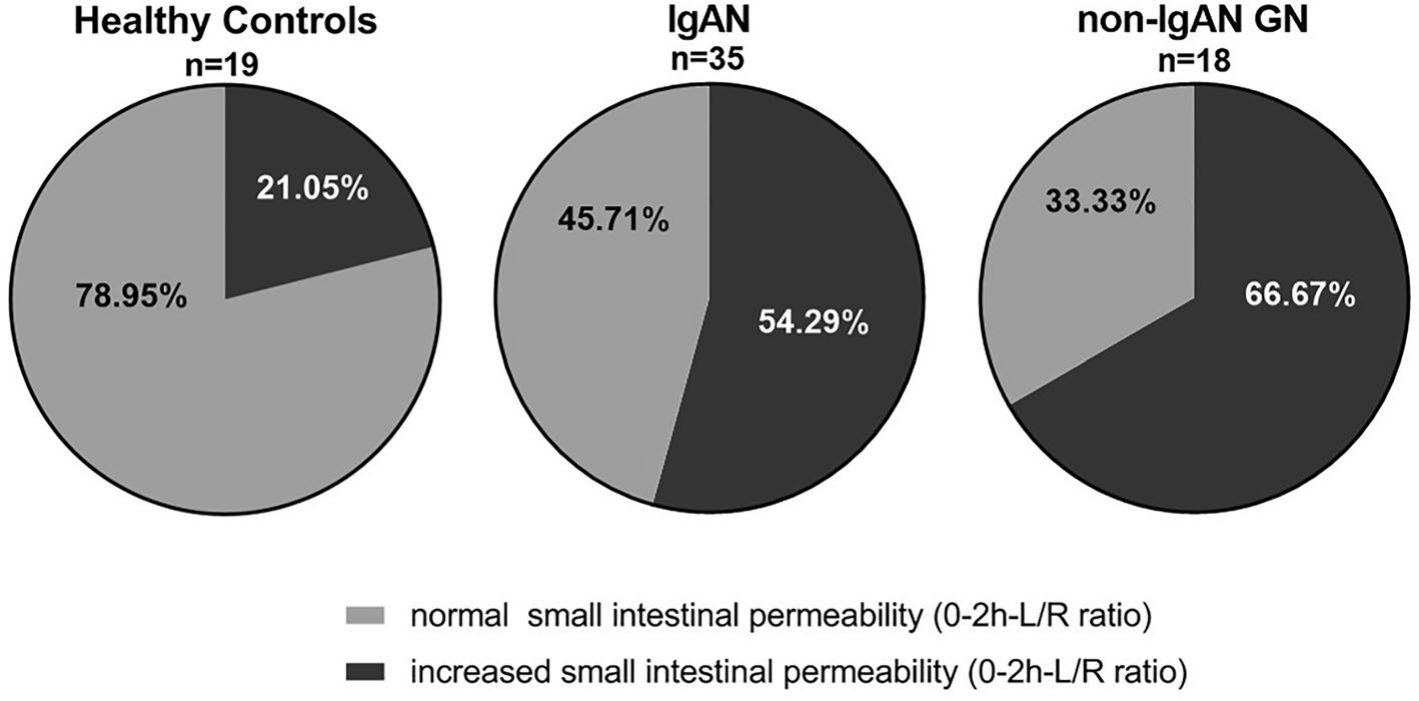
Proportions of normal and increased small intestinal permeability. Percentage of healthy controls, IgAN and non-IgAN GN patients with normal or increased small intestinal permeability defined by values ≥ or < 95%-CI of the 0–2 h-lactulose/l-rhamnose (L/R) ratio in healthy individuals

Fifty-four percent of patients with IgAN and 67% of patients with non-IgAN GN displayed 0–2 h L/R ratios above the 95%-confidence interval (CI) of healthy individuals (Fig. 2). The number of individuals with 0–2 h L/R values above the 95%-CI of healthy individuals did not differ significantly between IgAN and non-IgAN GN patients (*p* = 0.56) but was significantly higher than in healthy individuals (IgAN vs. healthy controls: *p* = 0.02, non-IgAN vs. healthy controls: *p* = 0.01).

### Association between renal dysfunction, proteinuria and intestinal permeability indices

**Fig. 3:**
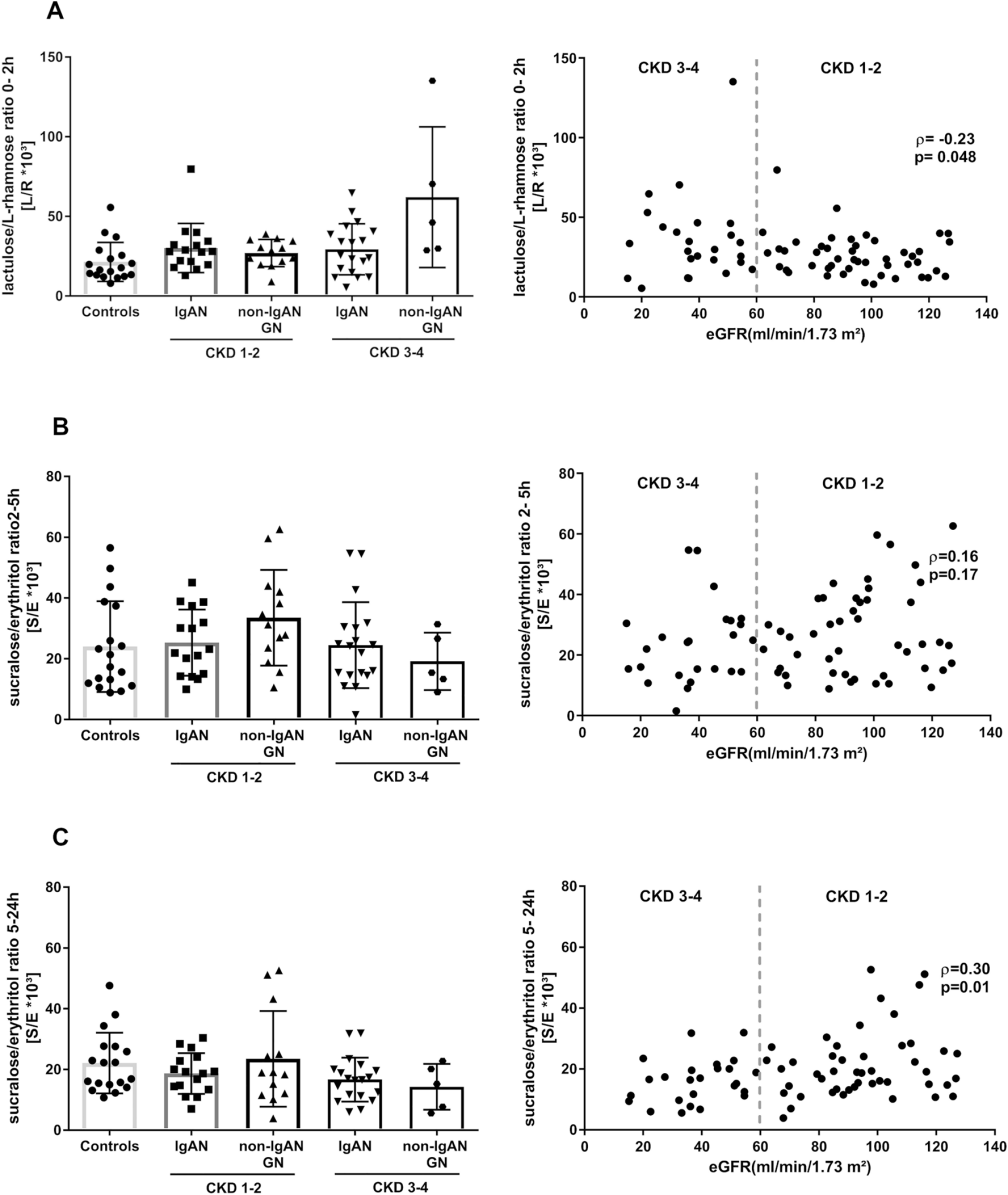
Association of CKD stages and gut permeability indices. **A** The 0–2 h-L/R ratio presented by CKD stages 1–2 and CKD stages 3–4 in patients with IgAN and non-IgAN GNs compared to healthy controls. The 0–2 h-L/R ratio did not change in IgAN patients with CKD Stages 3–4 compared to those in CKD stages 1–2. Patients with non-IgAN GNs in CKD stages 3–4 exhibited a non-significant increase in the mean 0–2 h-L/R ratio compared to non-IgAN GNs in CKD stages 1–2. The overall correlation of 0–2 h-L/R ratio and the patients’ eGFR was weak. **B** There were no changes in the 2–5 h-S/E ratio across all patient groups and CKD stages. The overall correlation of the 2–5 h-S/E ratio and the patients’ eGFR was weak. **C** There was no difference between the 5–24 h-S/E ratio between the patients with glomerular diseases and IgAN compared to healthy controls in CKD stages 1–2. The 5–24 h-S/E ratio of IgAN patients with CKD stages 1–2 was no different to those in CKD stages 3–4. Patients with other GNs showed a non-significant decrease of the 5-24 h- S/E ratio in CKD stages 3–4 compared to CKD stages 1–2. 0–2 h-L/R ratio, 0–2-h lactulose/l-rhamnose ratio; 2–5 h-S/E ratio, 2–5-h sucralose/erythritol (2–5 h-S/E) ratio; 5–24 h-S/E ratio, 5–24-h sucralose/erythritol ratio

**Fig. 4:**
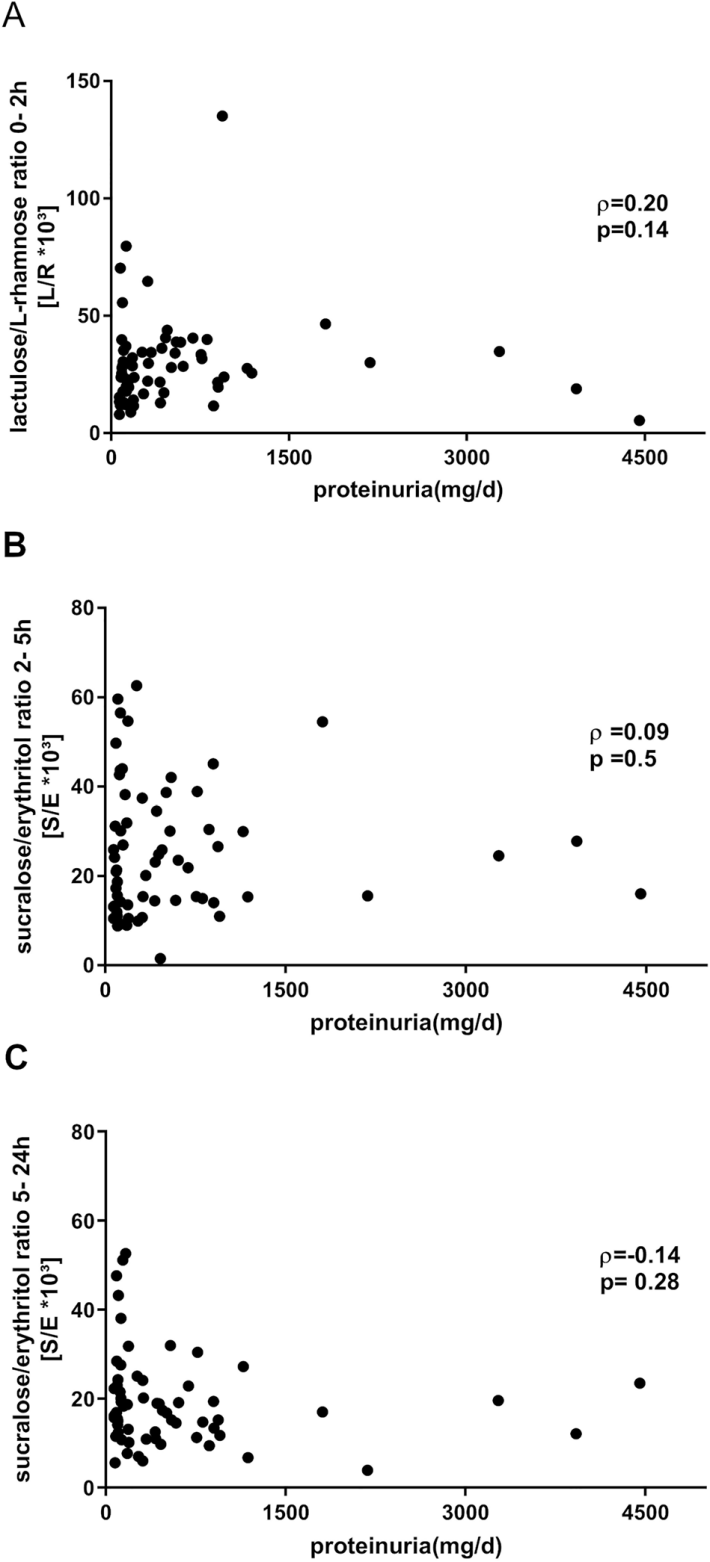
Correlation of proteinuria and gut permeability indices. **A**–**C** Correlation between proteinuria (mg/day) and either 0–2-h lactulose/l-rhamnose ratio (0–2 h- L/R ratio) (**A**), 2–5-h sucralose/erythritol (2–5 h-S/E) ratio (**B**) or 5–24-h sucralose/erythritol (5–24 h-S/E) ratio (**C**) of all patients (IgAN and non-IgAN GNs)

Next, we investigated the correlation between the individual eGFR levels and each of the permeability ratios of all participants. Overall, correlations between either eGFR or proteinuria and the three permeability ratios were weak. We only observed significant correlations between the eGFR and the 0–2 h L/R (*ρ* = − 0.23, *p* = 0.048) and the 5–24 h S/E ratio (*ρ* = 0.30, *p* = 0.01; Fig. 3). There were no significant correlations between proteinuria and any of the three permeability indices (Fig. 4A–C).

Subsequently, all GN patients were grouped by CKD stages 1/2 versus 3/4 and mean values for the three permeability indices are displayed in Table 3. Since the interaction between CKD stages and study groups was not included in the statistical model, we only looked at relative changes in a descriptive manner. We observed an average 1.4-fold increase in small intestinal permeability (i.e. 0–2 h L/R ratio) in IgAN patients in CKD stages 1–2 as compared to healthy controls, whereas the 0–2 h L/R ratio in non-IgAN GN patients did not differ from controls (Fig. 3A). Vice versa, in advanced CKD stages (CKD 3/4), IgAN patients did not differ from healthy controls, whereas small intestinal permeability increased on average by 2.9-fold in non-IgAN GN patients as compared to controls (Fig. 3A). For distal small intestinal and proximal colonic permeability (2–5 h S/E ratio) and colonic permeability (5–24 h S/E ratio), we observed no clear CKD-related differences (Fig. 3B, C).

**Table 3:** Permeability ratios for small intestinal permeability (0–2 h-L/R*10^3^) distal small and proximal colonic permeability (2–5 h-S/E*10^3^) and colonic permeability (5–24 h-S/E*10^3^) in healthy controls, IgAN and non-IgAN GN patients

	0–2 h-L/R*10^3^ ratio	2–5 h-S/E*10^3^ ratio	5–24 h-S/E*10^3^ ratio
Healthy controls (*n* = 19)	**21.66 ± 11.92**	**24.45 ± 14.63**	**21.56 ± 10.03**
IgAN (*n* = 35) *CKD 1–2 (n* = *16)* *CKD 3–4 (n* = *19)*	**29.74 ± 15.51** *30.17* ± *15.43* *29.38* ± *15.99*	**24.87 ± 12.60** *25.32* ± *10.91* *24.49* ± *14.15*	**17.59 ± 6.97** *18.68* ± *6.71* *16.68* ± *7.24*
Non-IgAN GNs (*n* = 18) *CKD 1–2 (n* = *13)* *CKD 3–4 (n* = *5)*	**36.77 ± 27.77**# *27.04* ± *8.51* *62.07* ± *44.19*	**29.53 ± 15.49** *33.51* ± *15.76* *19.16* ± *9.44*	**20.96 ± 14.36** *23.52* ± *15.74* *14.30* ± *7.53*

Bold values indicate statistically significant differences at the 5% significance level. Exact *p*-values of the comparisons can be found in Fig. 1

*L/R ratio* ratio between lactulose and l-rhamnose, *S/E ratio* ratio between sucralose and erythritol, *CKD* chronic kidney disease, *IgAN* IgA Nephropathy. Values are presented as means and standard deviations

#*p* < 0.05 as compared to healthy controls

## Discussion

It is well-established that disturbed intestinal barrier integrity may intensify the contact of environmental factors, food antigens, microbiota, and pathogenic microorganisms with the mucosal and systemic immune system. Subsequently immune reactions may be triggered and relapses or flares of (autoimmune) diseases involving the kidneys as target organs may occur [16–19]. This, for example, is evidenced by macrohematuria episodes in IgAN patients during mucosal infections. The involvement of the gut-kidney axis in IgAN pathophysiology has repeatedly been investigated for more than 30 years and led to the development of new treatment strategies [4, 20]. Pursuing an intervention within the mucosal immune system by using locally active budesonide, promising results have been obtained within the phase II Nefigan trial [11]. The subsequent phase III Nefigard Trial (NCT03643965) is currently ongoing.

Measuring intestinal permeability has been widely used to detect intestinal dysfunction in various diseases [21–23]. Nowadays, a very common method of measuring intestinal permeability in vivo is the lactulose/mannitol test, which has recently been extended to multi-sugar tests aiming to assess not only small intestinal permeability but also distal small intestinal and proximal colonic permeability as well as colon permeability in a time- and sugar-dependent manner [13, 14, 24]. The test involves a combination of di- and mono-saccharides that are ingested simultaneously followed by measurement of their urinary excretion. In case of a perturbed intestinal barrier, e.g., a higher rate of open tight junctions by epithelial disruption or inflammation, the paracellular transport, i.e., uptake of disaccharides, will increase. By contrast, monosaccharides are thought to be transported transcellularly and as such are used to correct for changes in other intestinal confounders, i.e., transit time. This is an advantage as compared to methods using radioactive ^51^Cr-EDTA alone. Using a single molecule might affect the measurement results as the transit of the molecule could be affected by inter-individual differences which might not directly be related to intestinal permeability (intestinal passage time, renal excretion) [25].

In IgAN patients, there is evidence of increased reactivity to dietary proteins from numerous nutritional studies (although not associated with overt dietary intolerance) including gluten, cow’s milk, and egg leading to a possible inflammatory reaction of the intestine with subsequent increase of intestinal permeability [11, 26–28]. Multiple findings (i.e. mucosal increase of inflammatory cells and cyclooxygenase 2 expression) are consistent with subclinical intestinal mucosal inflammation, and the degree of duodenal inflammation is significantly correlated with serum IgA and with the extent of proteinuria and hematuria [29]. However, other observations suggested that these pathological changes are not specific for IgAN: an analysis of duodenal mucosa from a mixed cohort of patients with IgAN and non-IgAN glomerular diseases revealed overall significantly increased numbers of intraepithelial T lymphocytes as compared to healthy controls, thus suggesting changes in intestinal permeability and a breakdown of oral tolerance as a more general phenomenon in such patients [30].

Most studies noted that the villous architecture of the duodenal small bowel in IgAN is macroscopically normal and no major histological changes are observed [31]. Consequently, we cannot expect changes in the intestinal permeability indices comparable to what is observed in diseases with severe disruption of the epithelial integrity as in celiac or Crohn’s disease. Nonetheless, an increase in small intestinal permeability has also been found in patients with other non-intestinal diseases such as acute exacerbated chronic obstructive pulmonary disease (COPD) with hypoxic respiratory failure or chronic heart failure [32, 33].

Hitherto, the role of intestinal permeability in IgAN patients is not yet completely understood due to differing methods (radioactive versus sugar-based) and differing duration of urine collection. Three studies assessed 24-h urinary excretion of radioactive ^51^Cr-EDTA normalized to creatinine clearance [6–8]. Using this method, increased intestinal permeability was documented for IgAN patients as compared to healthy controls, and increased intestinal permeability was associated with greater clinical activity of IgAN [6, 10, 34]. Of note, increased excretion rates for ^51^Cr-EDTA were not noted in all tested individuals. Prior studies that assessed intestinal permeability in IgAN patients by ingestion of lactulose or cellulose and mannitol were less encouraging and did not demonstrate any significant differences as compared to healthy controls [9, 10]. Possibly, these results were affected by the natural break-down of the sugars by colonic microbiota after 24 h of urine collection and were false negative.

We decided to apply a novel multi-sugar assay based on simultaneous ingestion of lactulose, rhamnose, sucralose and erythritol for the present study. This approach overcomes several problems of former IgAN studies in assessing gut permeability. By increasing the analytical sensitivity, the individual dosages of the ingested sugars that are known to induce increased gut motility were reduced. Additionally, the use of several different sugars allows to provide more accurate and site-specific information on small intestinal and colon permeability as sucralose and erythritol are not broken down by gut bacteria [14]. In our cohort, perturbations of the small intestinal permeability (0–2 h L/R ratio) were common in glomerular kidney diseases of different etiologies and did not distinguish IgAN from other glomerular diseases. As such, our findings are partly confirmatory of results obtained in previous studies [6, 8]. By contrast, elevated small intestinal permeability ratios did, if at all, only weakly correlate to established markers of a progressive disease course (i.e. lower eGFR or higher proteinuria). TThe number of individuals with 0–2 h L/R values above the 95%-CI of healthy individuals was significantly higher in IgAN patients than in healthy individuals. Yet, this was also true for patients with non-IgAN GNs. Taken together, these findings argue against the use of the permeability index as a marker of disease activity in IgAN.

Kidney function is thought to be a major confounder of measuring intestinal permeability indices by sugar-based assays. Both urinary flow as well as the glomerular filtration rate might influence the assay’s performance [35, 36]. Our correlation analysis only revealed a weak correlation between the eGFR and permeability markers. By contrast, the subgroup of patients with non-IgAN GNs showed an increase in small intestinal permeability in advanced CKD stages indicating that kidney function should still be considered a possible confounder when performing intestinal permeability studies in some glomerular diseases.

In our study cohort, only two IgAN patients received low-dose steroid therapy. Yet, in the study group of non-IgAN GNs, 15 patients received immunosuppressive treatment, mainly glucocorticoids with or without calcineurin inhibition. Available data from recent studies of intestinal permeability in patients after liver transplantation suggest that intestinal permeability is not substantially altered by the administration of calcineurin inhibitors [37, 38].

The effects of steroids on intestinal permeability are more complex based on the data available to date [39]. Several in vitro studies demonstrated improved intestinal permeability upon glucocorticoid treatment under “non-disease” conditions [39–41]. By contrast, other studies have shown increased intestinal permeability under high steroid doses in animal models of bowel inflammation, while steroids had no major damaging effect in non-colitic mice or lymphocyte transfer colitis [42]. In humans, the release of corticotropin-releasing hormone (CRH) might be involved in stress-induced increase of intestinal permeability[43]. By contrast, glucocorticoids also appear to have a positive effect on intestinal permeability in patients with inflammatory bowel disease [44–47]. Furthermore, local IgA production in the intestinal mucosa might also be influenced by steroid therapy [48–50], yet these effects on IgA production need further investigation. Taken together, steroid effects on the intestinal permeability might either be beneficial or deleterious depending on the underlying conditions. The present pilot study was not powered to investigate the effect of immunosuppressive treatment on intestinal permeability.

Our study has several limitations. First, the applied multi-sugar approach only analyzes epithelial integrity and not precisely whole intestinal barrier function. We cannot exclude that other mechanisms (i.e., IgA expression, the microbiome, the mucin layer, defensins, immunological changes) may lead to a pathologic exposure of environmental and pathogenic factors that in turn might deregulate mucosal IgA production. Additionally, our study was not powered to perform more complex analyses due to small sample size. Subgroup analyses of each glomerular disease were not considered meaningful for the same reason.

In conclusion, the majority of patients with IgAN and non-IgAN glomerulopathies exhibited increased small intestinal permeability. Along with the beneficial antiproteinuric effects of budesonide observed in the randomized Nefigan trial [11], our data indicate that a dysregulated intestinal barrier function can account for the development of IgAN in some patients. However, at the same time the present study suggests that disturbed intestinal permeability is common in patients with glomerular diseases and not specific for IgAN.
